# Self-calibration of C-arm imaging system using interventional instruments during an intracranial biplane angiography

**DOI:** 10.1007/s11548-022-02580-9

**Published:** 2022-03-12

**Authors:** Negar Chabi, Domenico Iuso, Oliver Beuing, Bernhard Preim, Sylvia Saalfeld

**Affiliations:** 1 Faculty of Computer Science, Otto-von-Guericke University Magdeburg, Universitätsplatz 2, 39106 Magdeburg, Germany; 2 Imec-Vision Lab, University of Antwerp, Universiteitsplein 1, 2610 Antwerp, Belgium; 3 Department of Radiology, AMEOS Hospital Bernburg, Kustrenaer Str. 98, 06406 Bernburg, Germany; 4 Forschungscampus STIMULATE, Magdeburg, Germany

**Keywords:** Self-calibration, Biplane X-ray imaging system, Perspective projection, Digital subtraction angiography (DSA)

## Abstract

****Purpose**:**

To create an accurate 3D reconstruction of the vascular trees, it is necessary to know the exact geometrical parameters of the angiographic imaging system. Many previous studies used vascular structures to estimate the system’s exact geometry. However, utilizing interventional devices and their relative features may be less challenging, as they are unique in different views. We present a semi-automatic self-calibration approach considering the markers attached to the interventional instruments to estimate the accurate geometry of a biplane X-ray angiography system for neuroradiologic use.

****Methods**:**

A novel approach is proposed to detect and segment the markers using machine learning classification, a combination of support vector machine and boosted tree. Then, these markers are considered as reference points to optimize the acquisition geometry iteratively.

****Results**:**

The method is evaluated on four clinical datasets and three pairs of phantom angiograms. The mean and standard deviation of backprojection error for the catheter or guidewire before and after self-calibration are $7.13\pm 6.47$ mm and $0.10\pm 0.06$ mm, respectively. The mean and standard deviation of the 3D root-mean-square error (RMSE) for some markers in the phantom reduced from $0.51\pm 0.11$ to $0.31\pm 0.08$ mm.

****Conclusion**:**

A semi-automatic approach to estimate the accurate geometry of the C-arm system was presented. Results show the reduction in the 2D backprojection error as well as the 3D RMSE after using our proposed self-calibration technique. This approach is essential for 3D reconstruction of the vascular trees or post-processing techniques of angiography systems that rely on accurate geometry parameters.

## Introduction

Biplane angiography has found increasing use in minimally invasive endovascular interventions to treat different types of aneurysms via coiling or stent placement. However, X-ray projection images lack 3D information of the vascular structures. This can be compensated for with the 3D reconstruction of vessels from a series (or a pair) of digital X-ray images. 3D reconstruction of the structures is of great clinical and diagnostic importance, since it allows physicians to examine the complex arterial network and to assess disease-induced changes in the vascular structure in three dimensions.

**Fig. 1:**
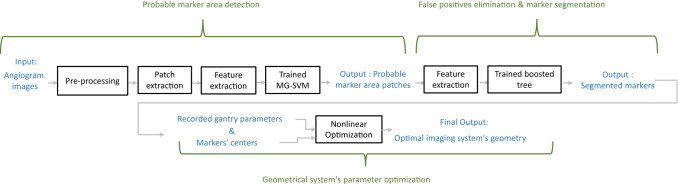
Our proposed self-calibration method pipeline includes marker detection and segmentation part plus the imaging system’s parameter optimization

To reliably reconstruct vascular structures, the exact geometry of the system, including the rotation and translation parameters that relate the two projection views, is needed for each configuration of the C-arm. Although DICOM image files contain angiographic system parameters, projection matrices directly derived from those parameters may not accurately describe the spatial relationship between the two views. Some main reasons are [1]:

1. The recorded gantry parameters may not exactly define the orientations of the C-arm
2. Unknown parameters such as skew parameter and principal point coordinate

An accurate geometry description of the imaging system is obtained through a calibration procedure using a specific calibration phantom or with self-calibration approaches. Although using a calibration phantom to determine the exact geometry of the system provides reliable results, it is not well suited for use in an intervention context due to its interference with the already complex clinical setting [2, 3].

On the other hand, self-calibration techniques, which use a set of reference points to iteratively optimize the projection parameters, impose a slight change to the clinical workflow.

Vascular features, such as bifurcations, are generally used to estimate the geometrical parameters of an angiographic system, while they may be too few or nonexistent in some clinical conditions [3–5]. For instance, acute proximal total coronary obstruction or carotid and vertebral vasculature may have few or even no bifurcations or identifiable corresponding points. Moreover, in calibration based on vascular centerlines, the epipolar lines intersect more than one centerline in most cases, which may increase the computations [1, 5, 6]. Therefore, interventional tools, such as guidewires, catheters, or stents with adhered radio-opaque markers, are ideal for calculating the system geometry [2]. The uniqueness of these devices may ease the finding of correspondences in two views compared to the search for corresponding vascular features. Interventional devices have rarely been used to optimize the system geometry [2].

We propose a semi-automatic approach to compute the more accurate geometrical parameters of the system, considering corresponding catheter or guidewire opaque markers in two views as reference points. The parameters are initialized considering the gantry recorded information, and an iterative nonlinear optimization is used to compute the parameters. To detect and segment the adhered markers in projection views, a novel machine learning approach is presented.

## Material and methods

The pipeline of our self-calibration approach is presented in Fig. 1.

### Data acquisition

Clinical biplane X-ray angiograms were acquired in intracranial endovascular interventions with an Artis Q (Siemens Healthineers, Forchheim, Germany) to treat vascular pathologies such as an aneurysm by coiling or stent placement. Images of a vascular silicon phantom with an aneurysm-like structure were acquired at 70 kV with the Siemens Artis Zeego angiography system (Siemens Healthineers, Erlangen, Germany).

### Radio-opaque marker detection and segmentation

Interventional instruments including catheters, guidewires, stents and flow diverters are equipped with radio-opaque markers that are typically made of gold and platinum to provide high visibility under fluoroscopy. We propose a novel method composed of two successive classifications to localize opaque markers. Therefore, they facilitate the detection of these devices. The first classifier aims to detect probable marker areas (Fig. 1). During this step, some marker regions may be missed, and at the same time, many false positives (FP) occur. We aim to select the classifier that has fewer false negatives (FN). In the second step, our goal is to remove FPs and to segment the markers.

To train the classification model for the 1st stage, four patient datasets, which include 30 images in total, were used. Then, patches with a size of $20 \times 20$ pixels or $3.08 \times 3.08$ mm were extracted from the normalized original 2D angiography images and their respective pre-processed image. This size is selected to include even the largest markers. For pre-processing, a bottom hat filter was used [7]. This filter enables background removal while keeping dark objects which are smaller than a specified structural element. Patches that include the whole markers are considered marker areas, while patches that include parts of the markers are discarded from the training set, and all other remaining patches from other parts of the image are considered as non-marker patches. The whole number of patches used to train the first stage classifiers includes 310 marker patches and 100k non-marker patches selected randomly among all non-marker patches.

From both the original and pre-processed images, 21 features were extracted. Due to the blob-like structure of the markers, blob analysis was used to generate features. Difference of Gaussian (DoG), Laplacian of Gaussian (LoG), and two types of the Determinant of Hessian (DoH) were computed for all the extracted patches [8]. The Hessian matrix for the first type of DoH is the directional gradient of the input, while the Hessian matrix for the second type of DoH is the second derivative of the Gaussian of the input. Then, their relative statistical features including mean and standard deviation were computed. The average and standard deviation value of the patches from the bottom hat image and the average, minimum and standard deviation of the patches extracted from the original image were added to the set of features too.

Different types of classifiers and different combinations including a variety of SVM, Naive Bayes, discriminant analysis, and Boosted Trees classifiers were trained [9]. Amongst them, the combination of medium Gaussian support vector machine (MG-SVM) for the marker detection part and Boosted Tree classifier for the segmentation stage provides the best results in terms of different criteria such as FNs and FPs.

**Fig. 2:**
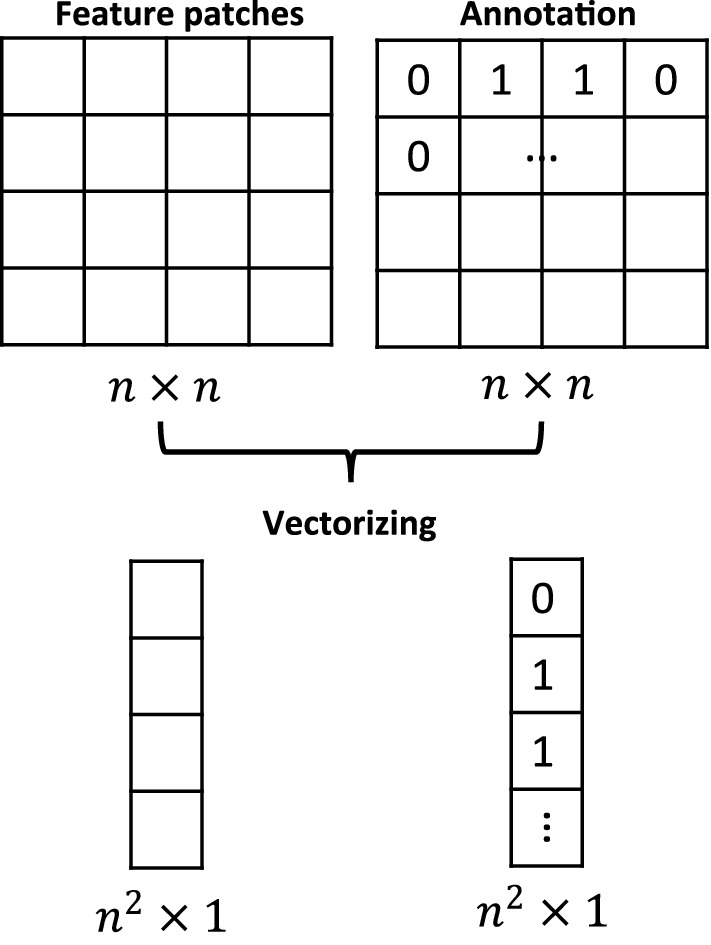
Vector-wise input features for second classification stage (segmentation)

For the first stage of classification, an MG-SVM model was trained. During finding probable marker areas in the first step, detected areas still contain many FPs. To get rid of these areas, another segmentation-based classification step was conducted. Detected patches from the previous stage were used as input for the second stage (namely the segmentation stage). The same type of features was used in the second stage to segment the markers and eliminate false positive regions. Features were fed vector-wise into the classification model (Fig. 2). A Boosted Tree classifier was trained by manual annotation of the markers, and for some patches to have a more accurate boundary for the markers, the multiplication of the thresholded original image patch and the thresholded DoH type one patch was used as ground truth. These thresholds were selected empirically. After segmentation, some areas that are no true markers, are still incorrectly segmented. Since the markers are necessarily located in the vessels, the results are further constrained by considering a vessel mask obtained by digital subtraction angiography (DSA).

### Prerequisites

#### Mathematical model of biplane angiography

**Fig. 3:**
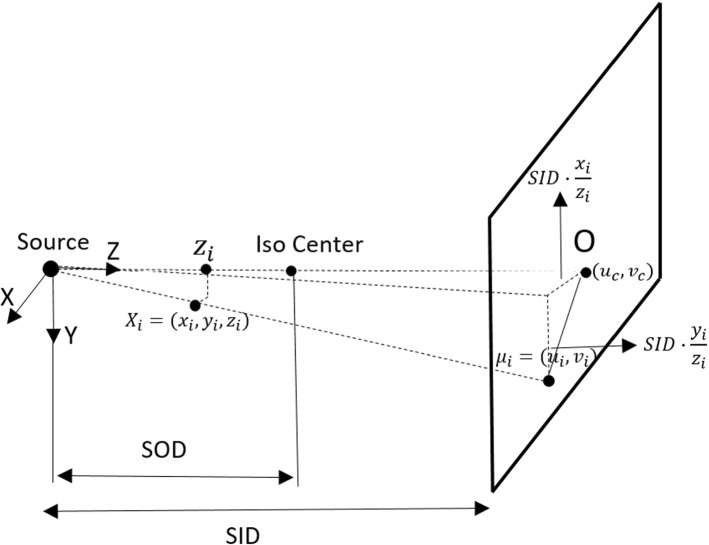
Angiographic projection geometry and its mathematical model [1]

X-ray angiographic acquisition is similar to the pinhole camera model (Fig. 3). The difference is that in the angiographic system, the image is magnified and not inverted. Projection of a specific object point in space $$X_{i}$$ onto the image plane is $$\mu _{i} =(u_{i},v_{i}) $$ . In Fig. 3, $$(u_{c},v_{c}) $$ refers to the principal point coordinate, SID and SOD denote the source to detector distance and the source to patient distance, respectively. Based on the perspective projection model, the projection process is represented as follows:

1 $$\begin{aligned} (x_{i},y_{i},z_{i})^{T}&\rightarrow \left( SID\cdot \frac{x_{i}}{z_{i}}+u_{c},SID\cdot \frac{y_{i}}{z_{i}}+v_{c}\right) ^{T}\nonumber \\&=(u_{i},v_{i})^{T} \end{aligned}$$

If we consider 3D space points and 2D points on the image plane in homogeneous coordinates as $X_{i}=(x_{i},y_{i},z_{i},1)^{T}$ and $\mu _{i}=(u_{i},v_{i},1)^{T}$, respectively, then the above equation can be represented in terms of projection matrix *P* for one view as below:

2 $$\begin{aligned} \mu _{i} = K\left[ I\mid 0\right] X_{i}=PX_{i} \end{aligned}$$

$$\begin{aligned} \textit{K} = \begin{bmatrix} \frac{SID_{j}}{pu} &{} \frac{SID_{j}}{pv}\cdot s &{} u_{c}\\ 0&{} \frac{SID_{j}}{pv} &{} v_{c}\\ 0 &{} 0 &{} 1 \end{bmatrix} \end{aligned}$$

where *I* is a $3\times 3$ identity matrix, 0 is a $3\times 1$ zero vector, and *K* represents the intrinsic camera parameters. *pu*, *pv* stand for image pixel spacing and *s* is the skew parameter. When there are two angiographic views, the geometric relationship between these views can be defined in terms of a $3\times 3$ rotation matrix *R* and a $3\times 1$ translation vector *T*. Finally, the projection of an object point $X_{i}$ is defined as follows (if we consider the source position of the first projection as the world origin):

3 $$\begin{aligned} \begin{aligned} x_{1,i} = P_{1}X_{i} = K_{1}[I\mid 0]X_{i} \\ x_{2,i} = P_{2}X_{i} = K_{2}[R\mid T]X_{i} \end{aligned} \end{aligned}$$

where *R* and *T* are described as follows:

4 $$\begin{aligned} R= & {} R_{x}(-\beta _{2})R_{y}(\alpha _{2})\cdot (R_{x}(-\beta _{1})R_{y}(\alpha _{1}))^{-1} \end{aligned}$$

5 $$\begin{aligned} T= & {} T_{2}-R\cdot T_{1} \end{aligned}$$

$X_{i}$ is computed by triangulation from two views, as described in [10]: Cross-product is performed on Eq. (3) to eliminate the homogeneous scale factor, then we have $x_{1,i}\times P_{1}X_{i} = 0$ and $x_{2,i}\times P_{2}X_{i} = 0$. Then, e.g., for the first view, the resulting equations are:

6 $$\begin{aligned} \begin{aligned} x (P_{1}^{3T}X_{i})-(P_{1}^{1T}X_{i}) = 0 \\ y (P_{1}^{3T}X_{i})-(P_{1}^{2T}X_{i}) = 0 \\ x (P_{1}^{2T}X_{i})-y(P_{1}^{1T}X_{i}) = 0 \end{aligned} \end{aligned}$$

Where $$x_{1} = \begin{pmatrix} x\\ y\\ 1 \end{pmatrix}$$ , $P_{1}^{iT}$ shows the $i^{th}$ row of $P_{1}$. The above equation is linear relative to $X_{i}$ (Eq.  (7)). As a result, if the projection matrices and correspondences in the two images are known, the space point $X_{i}$ can be accurately determined through these equations.

7 $$\begin{aligned} \underbrace{\begin{bmatrix} xP_{1}^{3T}-P_{1}^{1T}\\ yP_{1}^{3T}-P_{1}^{2T}\\ xP_{1}^{2T}-yP_{1}^{1T} \end{bmatrix}}_{A}\cdot X_{i} = 0 \end{aligned}$$

**Fig. 4:**
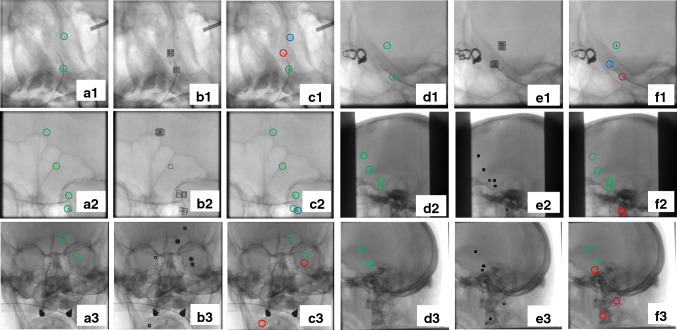
Results of marker detection and segmentation for test sets 1-3. Green, red and blue circles show the TPs, FPs, and FNs, respectively. (**a1**–**a3**). Original 1st view with the markers inside green circles, (**b1**–**b3**). Detected probable marker areas (1st view), (**c1**–**c3**). Marker segmentation results (1st view), (**d1**–**d3**). Original 2nd view with the markers inside green circles, (**e1**–**e3**). Detected probable marker areas (2nd view), (**f1**–**f3**). Marker segmentation results for the 2nd view

#### Iterative optimization algorithm

True correspondences, which are semi-automatically detected as described in Sect. 2.2, are used to find the accurate geometry of the system. Optimization is performed to determine both intrinsic and extrinsic parameters. Parameter values recorded by the system are used as initialization to start optimization. Then, for the intrinsic matrix, the skew parameter (*s*) is considered zero, and the principal point coordinate is initialized as the width and height of the image $\frac{C_{j}}{2}$ and $\frac{R_{j}}{2}$. $px_{j}$ and $py_{j}$ refer to the image pixel spacing.

8 $$\begin{aligned} K_{j}, \mid _{j = 1,2} = \begin{bmatrix} \frac{SID_{j}}{px_{j}} &{} s &{} \frac{C_{j}}{2}\\ 0 &{} \frac{SID_{j}}{py_{j}} &{} \frac{R_{j}}{2}\\ 0 &{} 0 &{} 1 \end{bmatrix} \end{aligned}$$

Initialization for the extrinsic parameters, including rotation matrix *R* and translation vector *T* is as follows: Based on the assumed coordinate system, rotation matrix and translation vector are computed as follows:

9 $$\begin{aligned} R= & {} R_{x}(-\beta _{2})R_{y}(\alpha _{2})\cdot (R_{x}(-\beta _{1})R_{y}(\alpha _{1}))^{-1} \end{aligned}$$

10 $$\begin{aligned} T= & {} T_{2}-R\cdot T_{1} \end{aligned}$$

**Table 1:** Performance comparison of different classifiers for marker detection

Classifiers\Criteria	TPR $(\frac{TP}{TP+FN})$	Precision$(\frac{TP}{TP+FP})$	Miss rate $(\frac{FN}{FN+TP})$	FDR $(\frac{FP}{FP+TP})$
MG-SVM	0.65	**0.56**	0.34	**0.44**
C-SVM	0.72	0.25	0.27	0.74
$Opt-NB^1$	0.81	0.01	0.18	0.98
RUSBT	**0.90**	0.02	**0.09**	0.97
BT	0.65	0.39	0.34	0.60

1Optimizable Naive Bayes (Opt-NB) Bold values indicates the best performance

**Table 2:** Performance comparison of different classifiers for marker segmentation

Classifiers\Criteria	TPR $(\frac{TP}{TP+FN})$	Precision$(\frac{TP}{TP+FP})$	Miss rate $(\frac{FN}{FN+TP})$	FDR $(\frac{FP}{FP+TP})$
MG-SVM	0.68	0.60	0.31	0.39
C-SVM	0.69	0.45	0.30	0.54
Opt-NB	**0.94**	0.21	**0.05**	0.78
RUSBT	0.86	0.41	0.13	0.58
BT	0.79	**0.62**	0.20	**0.37**

Bold values indicates the best performance

**Fig. 5:**
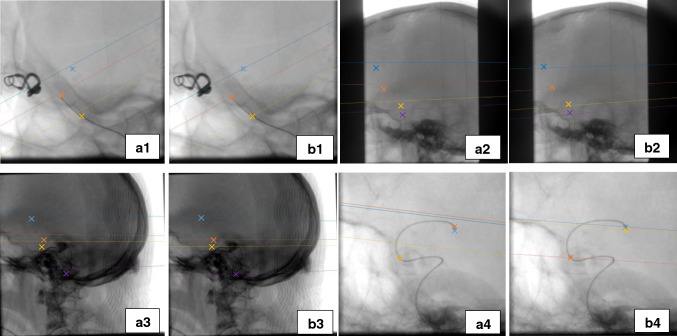
Epipolar lines before and after optimization; epipolar lines with the same color with the cross sign are relative to each other (Set 1-4), (**a1**–**a4**). Points and their relative epipolar lines before optimization, (**b1**–**b4**). Points and their relative epipolar lines after optimization

where $\alpha _{j}, \mid _{j = 1,2}$ refers to the LAO/RAO (left anterior oblique, right anterior oblique) angle, the rotation along the left, right-hand side of the patient and $\beta _{j}, \mid _{j = 1,2}$ refers to the CAU/CRA (caudal/cranial) rotation towards the patient for the two views. The goal of optimization is to minimize a cost function (Eq. (11)), here defined as the Euclidean distance between the position of the true correspondences and the backprojection of the 3D-reconstructed correspondences.

11 $$\begin{aligned}&{\text {*}}{argmin}_{P_{int}, P_{ext}} f(P_{int}, P_{ext})\nonumber \\&\quad ={\text {*}}{argmin}_{P_{int}, P_{ext}} \sum _{i = 1}^{n}d(x_{1,i}-\widehat{x}_{1,i})^{2}+d(x_{2,i}-\widehat{x}_{2,i})^{2} \end{aligned}$$

**Fig. 6:**
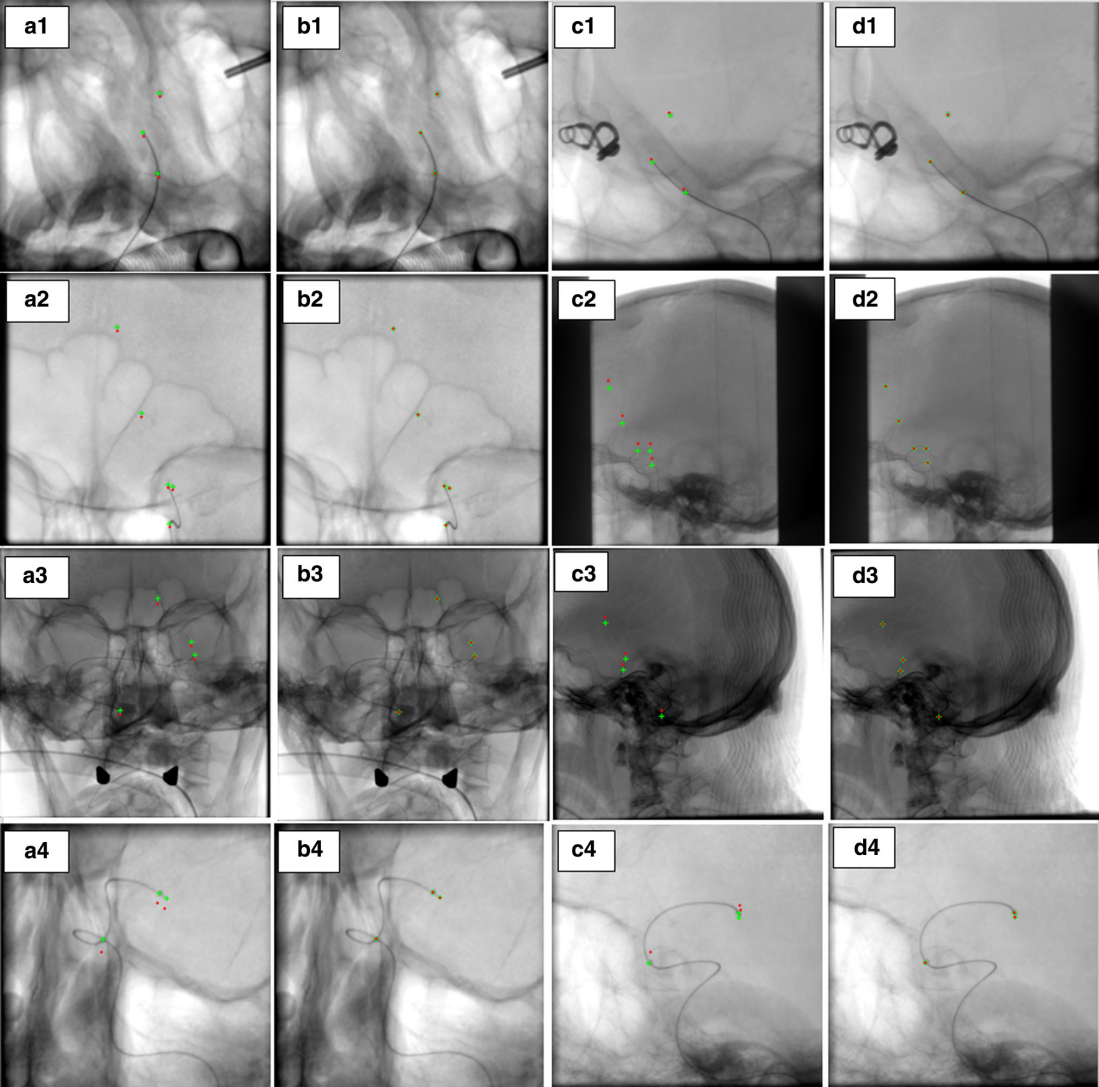
True correspondences backprojection before and after geometrical parameter optimization (Set 1-4), (**a1**–**a4**). 1st view before optimization, (**b1**–**b4**). 1st view after optimization, (**c1**–**c4**). 2nd view before optimization, (**d1**–**d4**). 2nd view after optimization

where *d* denotes the Euclidean distance between two points. $x_{j,i}$ and $\widehat{x_{j,i}}$, $\mid _{j =1,2, i = 1:n}$ are the reference points (extracted correspondences) and their relative backprojection of the 3D reconstructed reference points in two views, respectively, and *n* is the number of reference points. To minimize Eq. (11), an optimization algorithm, which is well suited for nonlinear least-square optimization, called the Levenberg–Marquardt algorithm, was used [11].

## Experimental results

In this section, the performance of our technique on routine clinical angiograms and phantom angiograms is evaluated. First, results of marker detection and segmentation for three test sets are presented in Fig. 4.

**Table 3:** Geometrical system parameters before and after self-calibration

Parameters	Parameter values (Set 1)		Parameter values (Set 2)	
	Before calibration	After calibration	Before calibration	After calibration
$\alpha _{1}^1$	-22.90	-21.92	-5.90	-3.41
$\alpha _{2}^1$	-107.80	-108.77	-93.20	-95.60
$\beta _{1}^1$	27.60	25.15	1.40	3.21
$\beta _{2}^1$	-16.00	-15.66	-0.20	-3.64
$SOD_{1}^2$	715.23	716.44	745.97	743.70
$SOD_{2}^2$	750.00	748.84	749.99	752.25
$SID_{1}^2$	1189.00	1188.24	1026.00	1027.66
$SID_{2}^2$	1207.00	1207.73	1131.00	1129.35

1In degree

1In mm

**Table 4:** Computed error based on Euclidean distance for the markers (mm)

1st or 2nd projection	Test set 1		Test set 2		Test set 3		Set 4	
	1st	2nd	1st	2nd	1st	2nd	1st	2nd
Before self-calibration	5.38	4.84	4.78	31.95	13.69	22.97	15.39	13.46
After self-calibration	0	0	0.02	0.16	0	0	0.24	0.20

During marker detection, the classifier most likely recognizes other marker-like structures such as the tip of the catheter or guidewire, and the location where the catheter or guidewire bends as marker areas leading to FPs. In the next stage (third and sixth column of Fig. 4), to limit the FPs and segment the markers, another classifier was trained. Different types of classifiers with different combinations were trained for both stages. Regions that were truly detected and segmented are enclosed by a green circle (TPs), regions that are wrongly detected and segmented as markers (FPs) are shown inside red circles, and regions that are missed and wrongly detected as non-marker (FNs) are enclosed by a blue circle. All other parts of the image that are not segmented, are truly classified as true negatives, which are not necessarily quantified.

The overall results of six test sets (including nine images) for some specific classifiers are listed in Tables 1 and 2 [12]. Amongst them, MG-SVM for the first classifier and BT for the second classifier perform the best in terms of the highest precision and lowest false discovery rate (FDR) (Tables 1 and 2). Therefore, a combination of MG-SVM and BT classifier was selected for the detection and segmentation part, respectively.

The centers of connected components are used as reference points for the next step (geometrical parameter optimization). The user corrects the results of segmentation by confirming the true reference points and removing FPs. Because the catheter and guidewire tip are almost identical to the markers, the method most likely detects these points as well, which can be kept as true correspondences.

The performance of geometrical parameter optimization is evaluated on four clinical sets (including three of test sets and one of the training sets (mentioned as set 4) of the first part (marker detection and segmentation)). Figure 5 shows epipolar lines before and after calibration for some true correspondences (including markers or tip of catheter or guidewire).

**Fig. 7:**
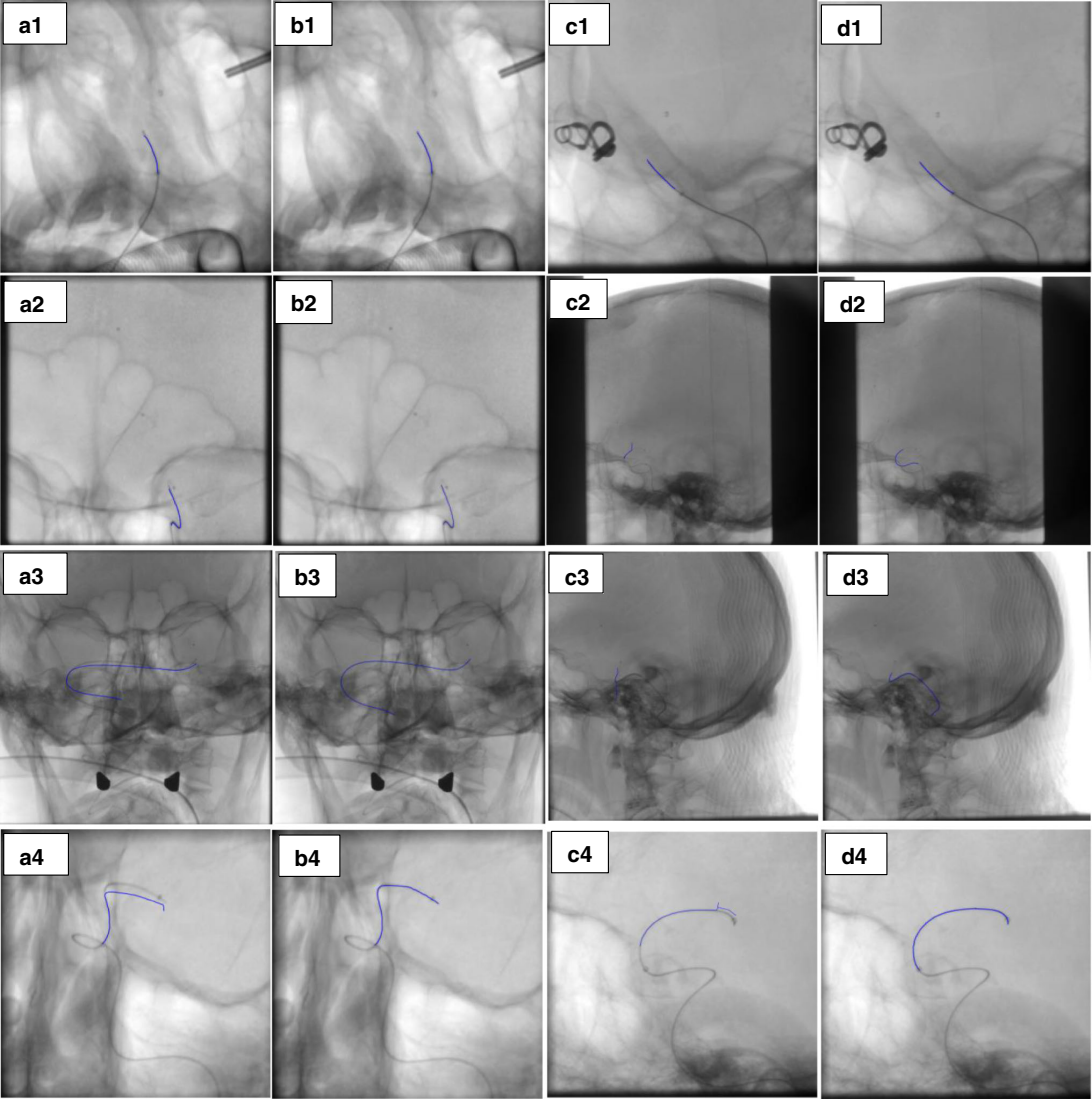
Backprojection of the catheter and guidewire before and after self-calibration, (**a1**–**a4**). 1st view before self-calibration, (**b1**–**b4**). 1st view after self-calibration, (**c1**–**c4**). 2nd view before self-calibration, (**d1**–**d4**). 2nd view after self-calibration

The epipolar constraint refers to the relationship between the epipolar plane and the epipolar line. The epipolar plane between two views is the plane passing through the point of interest on one of the image planes and the two source positions. The epipolar line is the intersection of this plane and another image plane. According to the epipolar constraint, given a point in the first view, its projection on another view must lie on its relative epipolar line on that view. As can be seen, upon self-calibration, the epipolar lines intersect accurately with the projected markers in the second view, while before parameter optimization they have a distance to their respective position, which is shown by crosses with the same color.

To better evaluate how optimizing the geometrical parameters affects the 3D reconstruction, we provide the results of backprojection for some known true correspondences (including markers and the tip of catheter and guidewire) in both views. As shown in Fig. 6, backprojections for true correspondences before calibration do not well coincide with their real position in both views, while after calibration their backprojections coincide very well with their real projection. Green pluses refer to the true correspondences (ground truth), and red pluses show the back projection of the 3D reconstructed points by the initial parameters (before optimization) and with the optimized parameter after calibration.

As a quantitative result, the distance error (in Euclidean distance) between real positions of the known correspondences and the backprojection of their relative computed 3D reconstructed position is presented in Table 4. The results are provided in mm.

Furthermore, Table 3 lists both the initial values for some parameters to be optimized, such as (LAO/RAO angle, CAUD/CRAN angle, SID and SOD for both projection views) and their respective values after self-calibration. As can be seen, parameters do not deviate a lot from their initial values and the changes are sensible.

**Table 5:** Computed error based on Euclidean distance for the catheter or guidewire (mm)

	Test set 1	Test set 2	Test set 3	Set 4
Before self-calibration	1.16	5.94	17.97	3.47
After self-calibration	0.06	0.06	0.14	0.12

**Table 6:** 3D error in mm. RMSE between the ground truth and the 3D reconstructed points before and after self-calibration

	Set 1	Set 2	Set 3
Before self-calibration	0.52 ± 0.13	0.64 ± 0.19	0.36 ± 0.10
After self-calibration	0.41 ± 0.14	0.34 ± 0.21	0.19 ± 0.20

Figure 7 shows the results of backprojection for the guidewire and catheter in projection views before and after calibration. 100 equal distance points are considered on the catheter or guidewire in the first view, and the corresponding epipolar lines are drawn in the second view using the initial and optimized parameters. Corresponding points of the catheter or guidewire in the second view are selected considering the proximity to the epipolar lines or the intersection with the epipolar lines as well as a moving order which starts from a specific starting point and ends in a specific point on the device (starting point and end point could be the device tip and the attached marker, respectively). These correspondences are used to reconstruct the device in 3D based on the initial and optimized parameters. Then, the backprojection of the 3D reconstructed device is computed, and a polynomial is fitted to the computed backprojection points to have the results in the form of the curve in both views. Figure 7 indicates that the backprojection of the catheter or guidewire after self-calibration coincides very well with their real projections. Furthermore, to better evaluate the method’s performance, RMSE between the real projection of the device and its backprojection before and after self-calibration is shown in Table 5. We conclude that the optimization does not only fit some limited reference points, but also works for other structures in the image.

To validate the method in 3D space, we used three different pairs of angiograms from a phantom acquired with MeVisLab with specific parameters [13].

Projection angles have been changed by $\pm {1^{\circ }}$ (a slight change that may occur in real cases as well) alternatively to $\alpha 1$, $\alpha 2$, $\beta 1$, $\beta 2$ for a total of 8 different combinations.

Then, the RMSE between the ground truth coordinates and the coordinates before and after self-calibration is computed (Table 6).

**Fig. 8:**
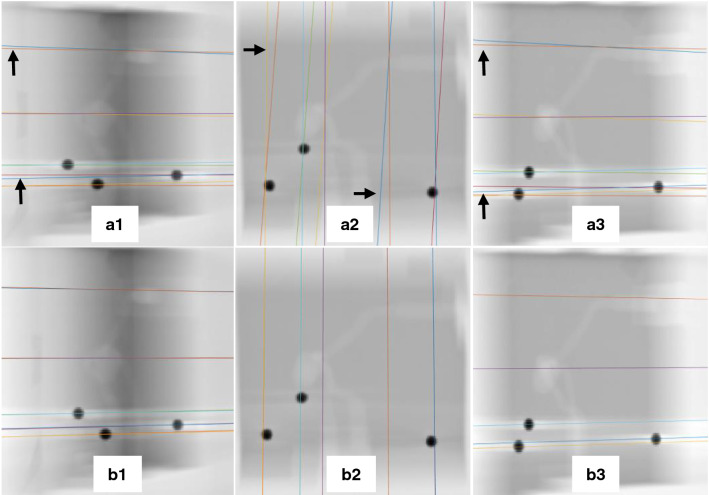
Comparison between ground truth epipolar lines, before self-calibration (with deviated parameters) and after self-calibration, (**a1**–**a3**). Comparison between ground truth epipolar lines and epipolar lines before calibration, (**b1**–**b3**). Comparison between ground truth epipolar lines and epipolar lines after calibration

## Discussion

The 3D reconstruction of digital subtraction angiography improves the safety of endovascular embolization of intracranial aneurysms. The importance and value of 3D-DSA is discussed in-depth in the study by Missler et al. [14]. In their study, 3D-DSA was generated using rotational DSA and the calculations are done based on calibrated images obtained using several phantoms. In 89% of the cases, 3D-DSA provided additional information about the aneurysm anatomy and 43% of these cases were better visualized on 3D-DSA images than on standard DSA images.

We proposed a technique to calibrate the imaging system using the adhered markers on the stent, catheter, guidewire, or their tips as correspondences instead of vascular features. We proposed a marker detection technique based on machine learning, which works well for markers that may not always be paired. Previous studies such as Schoonenberg et al. [15] are specifically suitable for marker couple detection in calibrated rotational angiography. Wang et al. [16] proposed a technique to localize the balloon marker pair and the guidewire between them with the aim of application for 3D stent reconstruction. This method is suitable for pair marker detection for balloon-mounted stents.

Therefore, for opaque markers that are not necessarily pairwise like in our study, other techniques have to be designed. Accordingly, in our previous study [8], we proposed a general approach to detect stent and catheter markers. The main differences between our previous and our current approach, are as follows: different pre-processing techniques were used, in the previous technique, adaptive thresholding was used to detect probable marker areas and its performance highly depends on its sensitivity value, which finally negatively affected the generalizability of the method. Then, to differentiate marker areas from non-marker areas, classification was used. While in the current approach, both detection and segmentation were done using machine learning classification models.

According to Tables 1 and 2, MG-SVM for the first classifier and BT for the second stage perform the best in terms of Precision and FDR, and at the same time, TPR pertains at $65\%$ and $79\%$ for the first and second stage classification, respectively. While other classifiers with higher TPR generate a high number of false positives, which is obvious based on their precision and FDR value.

Most of the previous studies rely on bifurcation points to self-calibrate the imaging system [3, 5, 17]. However, in some clinical situations, there may be a lack of or not enough bifurcation points available to refine the system’s geometrical parameters. Some studies utilized centerline corresponding to correct the system geometry, which needs exact corresponding vessels or vessel branches to be specified in different views [1, 6].

Online calibration was implemented by Vachon et al. [2] using elongated shaped instrumentations, such as a catheter or guidewire inserted into the patient during treatment for pulmonary stenosis. They used the segmented guidewire to correct the correspondences in the 2nd view. Marked beads were used to correct the epipolar lines in the 2nd projection. The beads are determined manually, and the guidewire segmentation is corrected manually. The limitation of this study is that elongated structure segmentation is a prerequisite, which needs to be corrected manually. Furthermore, the marked beads were manually annotated.

If there are enough markers in addition to the tip of the catheter and guidewire, these points can be considered as reference points for modifying the recorded gantry parameters. Our proposed semi-automatic technique relies on the markers attached to the guidewire or catheter to modify the geometrical parameters of the system. This is very desirable because marked guidewires, catheters or stents are frequently used during intracranial endovascular interventions.

The proposed technique also improves accuracy in terms of epipolar lines, which coincides accurately with the projection of the markers in the second view (Fig. 5). Furthermore, the improved accuracy is confirmed by computing the Euclidean distance of the markers with their projections before (4.84–31.95 mm) and after self-calibration (0–.24 mm) (Table 4). The comparison of the projection of the markers and the tip of catheter and guidewire with the initial values before and after self-calibration is well shown in Fig. 6. The backprojection of the markers with the optimized parameters coincides well with their real position in both views.

This improved accuracy is further confirmed for catheter and guidewire, and it demonstrates that the optimization of the parameters not only works for the markers, but also for other objects in the projection images, the backprojection error for the catheter and guidewire reduced from $7.13\pm 6.47$ mm before self-calibration to $0.10\pm 0.06$ mm after self-calibration (Fig. 7 and Table 5).

Therefore, we expect that this approach also works for other structures in the projections, such as vascular trees.

Validation in 3D space was done using a phantom. According to the results in Table 6, it can be seen that the 3D RMSE error reduced from $[0.36 - 0.64]$ mm with mean and STD of $0.51\pm 0.11$ mm before self-calibration to $[0.19 - 0.41]$ mm with mean and STD of $0.31\pm 0.08$ mm after self-calibration.

Furthermore, to visually assess the impact of self- calibration, the epipolar lines before and after self-calibration are drawn and compared with the ground truth epipolar lines (Fig. 8). It is clear that before self-calibration there exists deviation between the ground truth epipolar lines and epipolar lines before calibration (some deviations are indicated with black arrows) (Fig. 8 (a1-a3)), while this deviation is clearly diminished after self-calibration (Fig. 8 (b1-b3)).

The total average processing time, excluding the time needed for the confirmation of the markers for all three test sets, was 20.01*s*.

One of the main limitations of our approach is that the markers needed to be confirmed by the user and, in case of false-negative, the user should select the marker which causes errors imposed by the user and may negatively affect the accuracy of the method. In future work, we aim to alleviate this problem by making it fully automatic.

## Conclusion

This study presents a self-calibration approach for biplane angiography using known correspondences in both views, including the relative features of the interventional instrument such as catheter and guidewire. The radio-opaque markers attached to these devices as well as the tip of the catheter and guidewire can be used as reference correspondences to correct the geometrical parameters of the system. To detect and segment the markers, a novel machine learning approach is proposed. An iterative nonlinear optimization algorithm is employed to optimize the projection parameters. Geometrical information provided in the DICOM header is considered as a reasonable initialization for the optimization process. This approach corrects the geometrical parameters of the system with a slight change to the regular clinical workflow.

Future steps may include considering not only the adhered opaque markers or the device tip, but also the centerlines of the catheter and guidewire to correct the system geometry in case of insufficient correspondences or insufficient accuracy. Furthermore, the current approach could be improved to a fully automatic self-calibration if the marked catheter or guidewire is correctly segmented. Moreover, this approach may be extended for other applications, including other endovascular procedures with the presence of marked intervention instruments.
